# Book Notices

**Published:** 1868-10

**Authors:** 


### Book Notices.

Vesico-Vaginal Fistula from Parturition and other Causes: With Cases of Recto-Vaginal Fistula. By THOMAS ADDIS EMMET, M.D., Surgeon-in-Chief to the New York State Woman's Hospital, etc., etc. New York: William Wood & Co. 1868. For sale by W. B. Keen & Co.

This is an octavo volume of 250 pages, good paper, fair type, and good binding. It is a very interesting monograph on the subject of vesico-vaginal fistula; embracing the causes, nature, and best methods of treating this class of injuries. It contains cuts representing the various instruments used, the position of the fistula with the sutures introduced, and many illustrative cases. It is a valuable addition to our literature.

---

Diseases of Children. A Clinical Treatise based on Lectures delivered at the Hospital for Sick Children, London. By THOMAS HILLIER, M.D., London, Fellow of the Royal College of Physicians, Physician to the Hospital for Sick Children, etc., etc. Philadelphia: Lindsay & Blakiston. 1868. For sale by S. C. Griggs & Co. Price, $3.00.

This is a neatly-published octavo volume of 400 pages. The author treats of the following diseases:—Pneumonia; Lobar Pneumonia; Pleurisy; Rickets; Tuberculosis; Diphtheria; Acute Hydrocephalus and Meningeal Tubercle; Chronic Hydrocephalus, Tubercle of the Brain, and other Cerebral Affections; Pyæmia and Ottorrhœa; Chorea; Paralysis; Ascites; Scarlatina; Typhoid Fever; Skin Diseases; Epilepsy and Convulsions; with an Appendix giving formulæ for medicines in children. We have not had time to examine the work sufficient to express an opinion of its merits.

---

Atlas of Venereal Diseases. By A. CULLERIER, Surgeon to the Hospital Du Midi; Member of the Surgical Society of Paris, etc. Translated from the French, with Notes and Additions, by FREEMAN J. BUMSTEAD, M.D., Professor of Venereal Diseases in the College of Physicians and Surgeons of New York, etc. With about 150 beautifully-colored Figures on twenty-six Plates. Philadelphia: Henry C. Lea. 1868.

Part four of this valuable work is before us. One more part completes it. We gave a pretty full notice of its merits on the reception of the first parts, and now repeat the recommendation then expressed.

---

Archives de Physiologie Normale et Pathologique Publiées, par MM. BROWN-SEQUARD, CHARCOT, VULPIAN. No. 5, Septembre–Octobre, 1868. Paris: Victor Masson et Fils, 17 Place de L'École-de-Médicine.

This is the fifth number of the Archives of Physiology, both Normal and Pathological, published in Paris, by Brown-Séquard, Charcot, and Vulpian. It is a regular periodical issued every two months; and for such as read the French language readily, it is a most valuable work.

---

The Diseases Peculiar to Women, including Displacements of the Uterus. By HUGH L. HODGE, M.D., Emeritus Professor of Obstetrics and Diseases of Women and Children, in the University of Pennsylvania. With Illustrations. Second Edition, revised and enlarged. Philadelphia: Henry C. Lea. 1868.

This is an elegantly-published work of over 500 pages; containing the results of the study and observation of one who holds a high rank among the practitioners and teachers of Obstetrics and Diseases of Females, and who has been an active laborer for more than 40 years. This work is arranged in three parts:

The first treats of diseases of *Irritation;* embracing nervous irritation and its consequences, and irritable uterus.

The second treats of Displacements of the Uterus.

The third treats of "Diseases of Sedation;" under which title the author embraces Amenorrhœa and Chlorosis.

Without either criticising or commending all the views presented by the author, we say to our readers, the work is one that will amply repay them for a careful perusal. It is, indeed, one of the most valuable contributions to our medical literature. For sale by S. C. Griggs & Co., Chicago. Price, $4.00.

---

The Science and Practice of Medicine. By WILLIAM AITKEN, M.D., Edinburgh, Professor of Pathology in the Army Medical School. Second American, from the fifth, enlarged and carefully revised, London Edition. Adopting the new Nomenclature of the Royal College of Physicians of London. With large Additions, by MEREDITH CLYMER, M.D., Ex-Professor of the Institutes and Practice of Medicine in the University of New York; formerly Physician to the Philadelphia Hospital, etc., etc., etc. In two Volumes, with a Map, Lithographic Plate, and numerous Illustrations on Wood. Philadelphia: Lindsay & Blakiston. 1868. Price, $12.00.

On the appearance of the first American edition of this work, we commended it to our readers, as the most complete and reliable embodiment of the present status of Practical Medicine in Great Britain that had yet been published. The speedy call for a second edition shows that its merits have been extensively appreciated by the profession in this country. In the present edition, the author has added some new chapters, and the American Editor some two or three hundred pages of matter on important topics. It is altogether such a work as the intelligent practitioner will be reluctant to do without.

---

Criminal Abortion: Its Nature, its Evidence, and its Law. By HORATIO R. STORER, M.D., LL.B., Fellow of American Academy of Sciences, and late Professor of Obstetrics and Medical Jurisprudence in the Berkshire Medical College; and FRANKLIN FISKE HEARD. Boston: Little, Brown & Co. 1868.

This is a neatly-published octavo volume of 215 pages. It presents the statistics, nature, and medical jurisprudence of criminal abortion, in an interesting style, and with a fulness of detail, that makes it a valuable work for members of both the medical and legal professions. We know of no other source from which so much information, both medical and legal, can be obtained on this subject, as from this book.

---

GLYCERINE.—The solvent power of glycerine upon several substances commonly used in medicine and the arts, is as follows: One part of sulphur requires 2000 parts of glycerine; iodine, 100 part; red iodide of mercury, 340 parts; corrosive sublimate, 14 parts; sulphate of quinine, 48 parts; tannin, 6 parts; veratria, 96 parts; atropia, 50 parts; tartar emetic, 50 parts; iodide of sulphur, 60 parts; iodide of potassium, 3 parts; sulphide of potassium, 10 parts.—*Med. and Surg. Reporter.*

